# Towards Efficient Mobile M2M Communications: Survey and Open Challenges

**DOI:** 10.3390/s141019582

**Published:** 2014-10-20

**Authors:** Carlos Pereira, Ana Aguiar

**Affiliations:** Instituto de Telecomunicações, Faculty of Engineering, University of Porto, Porto 4200-465, Portugal; E-Mail: ana.aguiar@fe.up.pt

**Keywords:** Machine-to-Machine communications, mobile communications, gateways, cellular networks, wireless networks, wireless sensor networks, resource usage efficiency, smartphones, CoAP, MQTT

## Abstract

Machine-to-Machine (M2M) communications enable networked devices and services to exchange information and perform actions seamlessly without the need for human intervention. They are viewed as a key enabler of the Internet of Things (IoT) and ubiquitous applications, like mobile healthcare, telemetry, or intelligent transport systems. We survey existing work on mobile M2M communications, we identify open challenges that have a direct impact on performance and resource usage efficiency, especially the impact on energy efficiency, and we review techniques to improve communications. We review the ETSI standard and application protocols, and draw considerations on the impact of their use in constrained mobile devices. Nowadays, smartphones are equipped with a wide range of embedded sensors, with varied local and wide area connectivity capabilities, and thus they offer a unique opportunity to serve as mobile gateways for other more constrained devices with local connectivity. At the same time, they can gather context data about users and environment from the embedded sensors. These capabilities may be crucial for mobile M2M applications. Finally, in this paper, we consider a scenario where smartphones are used as gateways that collect and aggregate data from sensors in a cellular network. We conclude that, in order for their use to the feasible in terms of a normal depletion time of a smartphone's battery, it is a good advice to maximize the collection of data necessary to be transmitted from nearby sensors, and maximize the intervals between transmissions. More research is required to devise energy efficient transmission methods that enable the use of smartphones as mobile gateways.

## 1. Introduction

Machine-to-Machine (M2M) communications describe mechanisms, algorithms and technologies that enable networked devices, wireless and/or wired, and services to exchange information or control data seamlessly, without explicit human intervention. In this context, a machine is a device or piece of software, as opposed to a human.

M2M communications are expected to revolutionize telecommunication operators' business due to emergence of new networked applications, which will attract new clients and increase the data flowing in their networks, creating more billing opportunities. The Internet of Things (IoT) with its unlimited range of applications that rely on everyday objects becoming intelligent connected devices [1] is a major driver for M2M applications. The National Intelligence Council foresees that food packages, furniture, and even paper documents can be Internet nodes by 2025 [2]. IDC envisions 212 billion “things” by the end of 2020, where 30.1 billions are expected to be connected autonomously [3]. On the other hand, the sensing and connectivity capabilities of smartphones and their pervasiveness in people's lives make them critical pieces of this IoT and future applications. Thus, mobile M2M communications will certainly play a significant role in the M2M ecosystem and in cellular communications. The Cisco Visual Networking Index Global Mobile Data Traffic Forecast Update [4] estimates that in 2013 mobile M2M communications represented 1% of all mobile data with 4.9% of all connected devices, and by 2018 mobile M2M communications will represent 6% of all mobile data with 19.7% of all connected devices.

The staggering market of mobile M2M communications in vehicles leads Machina Research to expect an exponential growth in the number of vehicles with M2M connection capabilities in the next years [5], which demands further research in this area due to the mobile nature of vehicles. Vehicles can have embedded M2M devices, or users can carry devices, including smartphones, with M2M capabilities. This year in a survey, 80% of inquired drivers (5000 in total) expected that the car of the future provides them the same connected experience they have at home, at work, or on the move via their mobile phone [6]. Furthermore, more than 70% of them said they were interested in using or were already using internet services in their cars. The number of vehicles with built-in connectivity is predicted to increase from 10% of the overall market in 2013 to 90% by 2020 [7]. The same study predicts that the M2M market in the automotive sector will generate $422 billion by 2022, and services, including both connectivity and applications supported by it, shall account 88%.

Mobile M2M communications will revolutionise a very broad range of businesses like large sensing applications, intelligent transport systems, or monitoring and control applications, e.g., personal health monitoring, city automation, automotive systems, and smart grid [8–13]. Key challenges in mobile M2M will be the support of a large variety and diversity of devices, most of them with resource constraints (memory, processing, bandwidth, energy, etc.), and the traffic volume and traffic pattern they generate. M2M traffic differs from Human-to-Machine (H2M) or Human-to-Human (H2H) by the large number of short payload transactions [14]. The uplink traffic will be dominant in the first since M2M devices will rather be data generators, data resulted from monitoring or sensing application, than data consumers. Furthermore, the communication model in H2H is predominately request-response, while for M2M it is expected a partition between request-response and publish-subscribe.

Within mobile M2M communications, smartphones are bound to play a special role. They are equipped with wide environment and context sensors, as well as with multiple personal, local, and wide area communication capabilities, namely NFC, Bluetooth (Zigbee is expected in the near future), WiFi and several cellular technologies. They will likely be used as sensors themselves, and as data relays for other nearby devices with more limited connectivity, for example health sensors in a personal area network [15] or domotic sensors and actuators in a home automation environment [12]. The use of smartphones for transmission of sensing data, their own or relayed, can lead to faster battery depletion undesirable for the users, as they use their smartphones mainly for other purposes, like phone calls, SMS, or Web browsing. This article focuses on mobile M2M communications, considering their impact on devices with limited capabilities and constraints.

Other M2M communications' surveys focus mainly in home networks [16], 3GPP Long-Term Evolution (LTE) networks [17], service platforms [18], or data mining techniques [19]. Although wireless and cellular networks are commonly addressed in surveys and literature, we center the theme of this manuscript on mobile communications, and we emphasize the importance of resource usage efficiency, which can be further enhanced by the use of gateway devices. Additionally, we combine a concise review of standards and protocols related to M2M communications, which can serve as an initial starting point for researchers new to the field, or anyone that wants to have a general overview of the state of the art.

This document makes the following contributions:

- we survey the literature and structure current open challenges;
- we review standardization activities, architecture and common application-layer protocols, namely Constrained Application Protocol (CoAP) and Message Queuing Telemetry Transport (MQTT);
- we perform a preliminary study on the impact of using smartphones as entities that collect and aggregate information from sensors on their battery consumption.

This document is organized as follows: An extensive survey of the literature work and challenges are presented in Section 2. The reference architectures for M2M are presented in Section 3, and ETSI architecture is detailed in Section 4. In Section 5 we discuss M2M communication models and paradigms, and in Section 6 we detail two application protocols, CoAP and MQTT. Section 7 studies the feasibility of smartphones as gateways, and we conclude the document in Section 8.

## 2. Mobile M2M Literature Review

Mobile M2M communications face many technical challenges despite the promising benefits in terms of revenue opportunities and cost reductions in maintenance and resources [20]. M2M devices are usually small and inexpensive, introducing energy, bandwidth, computation, and storage constraints to communications [12]. The potential booming of M2M applications can exponentially increase the number and diversity of devices and traffic in the next years, which shall introduce further challenges to communications. Current mobile M2M communications research focuses on performance evaluation and improvement, either in terms of delay or resource usage efficiency. In this section, we survey relevant literature and structure current research areas. At the end of the section we point out some open challenges and directions for future work.

### 2.1. M2M Traffic

It is important to distinguish mobile M2M communications from mobile Human-based (H2H or H2M) communications. Small and infrequent data transmissions will be more common in M2M [17,21], and thus the knowledge developed for Human-based traffic, which is mostly bursty (web browsing), bulky (file transfer), or constant or variable bit rate streams (VoIP or video) can be difficult to apply directly to M2M. Laya *et al.* [17] mention that M2M and Human-based traffic differ further in traffic direction, since M2M traffic direction will be mainly uplink, while Human-based traffic is either balanced or mainly downlink. M2M applications will be duty-cycled and should have very short connection delay to guarantee fast access to the network when waken up, while Human-based applications tolerate longer connection delays but are very demanding once connections are established [17]. M2M applications might require very high priority with a detailed level of granularity due to the transmission of critical information, whereas priority for Human-based applications is mainly among applications for each user and not between different users [17]. Finally, M2M will have higher number of devices and may be required to operate for years or decades without maintenance, but users can recharge/replace batteries [17].

### 2.2. M2M Support in Wireless Networks

M2M devices using radio technologies will face well-known problems from wireless and cellular networks. Potential issues on the air interface including channel interference, channel quality fluctuation, and noise will be very common due to the multitude of devices and the characteristics of M2M traffic [12,21], and they can introduce coordination problems in the medium access. According to Lu *et al.* [22], reliability is critical for general acceptance of M2M, since unreliable processing, sensing, or transmission leads to false or lost data, and ultimately to M2M communications' failure from the user's perspective. Although end-to-end service reliability is still a challenge, it is being addressed by standardization efforts.

As the number of devices competing for the same channel increases, the number of simultaneous accesses will increase, and packet collisions, and signal interference in general, will be more common and result in more packet/data loss. Optimizing the uplink channel access and radio resource allocation is a way to achieve further improvement in performance and resource usage efficiency, avoiding constant transmission deferrals originated from the packet collision avoidance mechanisms and data loss originated from packet collisions, or providing general QoS guarantees. Gallego *et al.* [23] introduce contention-based MAC protocols for sensor-to-gateway communications in wireless M2M, and analyse them in terms of delay and energy efficiency. The authors consider an M2M wireless network composed of a large number of devices that periodically wake up their radio interfaces to transmit data to a coordinator, that is, to a gateway. Zhang *et al.* [12] propose a joint rate and admission control scheme for QoS provision in M2M communications, using an IEEE 802.11 network, by exploiting heterogeneous networks and accurate predictions of QoS. Wireless networks usually use solely collision avoidance mechanisms, which introduce well-known problems, such as the hidden node problem or the exposed node problem, that other networks do not face, such as cellular networks. Further work needs to be carried using wireless networks, in order to take advantage of the high data rate and low latency common in those networks.

Techniques that efficiently aggregate the data to be transmitted can be explored to further optimize bandwidth utilization and energy consumption in M2M communications. Two data aggregation schemes based on the Karhunen-Loève transform for M2M in a wireless network are proposed by Matamoros *et al.* [24]. Their system includes several sensors, one gateway, and one application server. The sensors transmit the data to the gateway, which transmits all the data to the application server. While gateway-to-application server communications use a reservation-based MAC protocol, the sensor-to-gateway communications use a contention-based MAC scheme and, thus, packet collisions may occur. They determine the optimal duration of the sensor-to-gateway and gateway-to-server transmission phases, in such a way that the best trade-off between the number of packet collisions and compression level from data aggregation is attained.

### 2.3. M2M Support in Cellular Networks

Nowadays cellular networks offer wide coverage areas, high data rate, and decreasing latency, and therefore they are a key enabler of M2M communications. The challenges associated with mass-scale M2M networks can be resumed to the multitude and diversity of devices, the scalable connectivity, and supporting of both legacy and new services and devices [25]. Marwat *et al.* [26] argue that, even in the presence of regular LTE traffic, mobile M2M traffic can not be considered negligible, and it can have a dramatic impact on the LTE network performance in terms of Quality of Service (QoS) and throughput.

Costantino *et al.* [27] evaluate the performance of an LTE gateway using CoAP and representative M2M traffic patterns and network configurations through simulations. The authors argue that traffic patterns depend very much on the single application considered, and, therefore, do not describe or justify their choices. The scenario consists of a single LTE cell where the evolved NodeB (eNB), the only mandatory node in the radio access network (RAN), serves one LTE M2M gateway and a variable number of terminals with traditional Internet traffic, called H2H User Equipments (UEs). The LTE M2M gateway, in turn, serves a variable number of smart objects. The results showed that LTE is sensitive to both intra-UE and inter-UE signal interference, which results in a high delay or packet loss when the number of smart objects served is greater than a few tens or the cell throughput approaches its limits. Tesanovic *et al.* [28] describe algorithms for device management to mitigate interference and device co-existence in LTE.

Similar to wireless networks, M2M communications for cellular networks can benefit from improvements on channel access or by introducing data aggregation techniques. A contention based uplink channel access for M2M in an LTE network is proposed by Zhou *et al.* [29]. With contention based access, UEs select resources randomly without indications from eNB, which saves signalling overhead and, hence, latency is reduced. Simulation results showed that a network coordinated random access stabilization scheme used to control the expected number of simultaneous access to a common random access channel (RACH) can effectively improve the access delay in LTE-Advanced [30].

Lo *et al.* [31] study the impact of data aggregation in M2M on throughput and delay in a cellular network. They motivate the use of an M2M relay as an M2M data aggregator to improve uplink transmission efficiency in LTE-Advanced due its overheads. They propose a tunnel-based aggregation scheme in which only M2M data units destined to the same tunnel exit point are aggregated at the tunnel's entry point, according to priority classes. The results show a significant reduction in protocol overheads. Furthermore, the results show that aggregation, as expected, increases the delay per unit in the delivery, but the global delay can rapidly decrease with the increase of M2M devices.

Transmission scheduling schemes can be introduced in mobile M2M communications to reduce delay or to achieve higher energy consumption efficiency. Yunoki *et al.* [32] achieves a delay reduction in a remote monitoring system by using a transmission scheduling scheme used in an Evolution-Data Optimized (EVDO) and in a Wideband Code Division Multiple Access (W-CDMA) networks. They achieve a probability of sensor status not reaching a monitoring center within a delay of 6 s lower than 10^−6^ compared to the probability of 10^−4^ for a best-effort effort scheme. This transmission scheduling scheme achieved more than 85% of average throughput compared to the best-effort scheme for the W-CDMA network, while having a similar performance for the EVDO network.

Pauls *et al.* [33] study the viability of using the General Packet Radio Service (GPRS) for a low data rate long-lasting battery powered operation of M2M devices. The authors evaluate optimizations of data transmission procedures to reduce the power consumption of GPRS connections, for transmitting small size and latency tolerant user data. For applications that require frequent transmissions, it is better that the devices are always turned on, but, for applications that do not require frequent transmissions, dramatic savings in power consumption (93%) can be obtained if the devices are turned off during the periods that do not transmit.

### 2.4. Energy Efficiency

Resource usage efficiency is one of the most important requirements for mobile M2M communications when using radio technologies, due to lower available bandwidth, higher link failure, and higher energy consumption. The amount of devices and the requisite that they might have to operate for many years with the same battery, or consuming the least possible energy, demands for energy efficiency in M2M communications [12,22]. Lu *et al.* [22] argue that M2M communications cannot be widely accepted as a promising communication technology, if energy efficiency is not met. In Section 7 we illustrate the importance of energy efficiency when using smartphones as mobile M2M gateways.

The concept of using a mobile M2M gateway device as an intermediary node to collect and process data from neighbouring sensors is approached by Wu *et al.* [25], who name it a smart M2M device, and Zhang *et al.* [12], who name it a cognitive gateway. Both works argue that connecting devices through a gateway should be preferred when they are sensitive to cost or power. The use of M2M gateways shall have a direct impact in the reduction of devices accessing and using the channels for communications, reducing interference and contention, and increasing the efficiency. Reducing the number of devices in networks also translates into easier to deploy and less complex transmission scheduling schemes, and eases the problem of the depletion of the pool of unallocated IP addresses.

New applications and business opportunities will come along with mobile M2M communications. For example, the innovative idea of using the already existing scheduled airliners as relays between ground devices and satellites, providing a new and complementary M2M infrastructure, is presented in [34]. Mobile M2M devices in airplanes can act as M2M gateways by forwarding data received from M2M ground terminals to satellites, and vice versa. With this approach, there is no need for M2M terminals in satellites to have a very powerful amplifier or large dish antennas to send or receive messages, and, thus, the operational costs should be lower. Furthermore, this solution addresses the challenge of connectivity, eventually relieving traffic from cellular networks. However, one should expect important challenges originated from transmission scheduling and mobility.

### 2.5. Device Mobility, Autonomy, and Security

Devices that are able to connect to multiple different networks will have significant signalling traffic overheads for vertical handovers. Furthermore, devices in vehicles might face constant vertical handovers originated from the vehicles' mobility. Discussion on the necessity of improving vertical handovers in M2M communications are presented in [35]. Kellokoski *et al.* [36] propose an energy efficient algorithm for vertical handovers between an IEEE 802.11 and a 3GPP network for M2M communications. The connectivity and cross-platform networking originated from the vehicles' mobility and positional distribution should also be a concern.

M2M communications should operate seamlessly without human intervention, and therefore self-configuration, self-management, and self-healing are important challenges [12]. Envisioned applications for mobile M2M communications require autonomous data collection and aggregation, transmission and distribution of aggregated data, and storing and reporting of information [37]. Furthermore, the total absence of, or limited, human intervention in many M2M applications can occasion physical attacks from malicious attackers, disrupting communications. Security for M2M communications is discussed in [22,25,37]. Security is a major aspect for, as an example, vehicular collision-avoidance applications [37] or for healthcare applications [15].

### 2.6. Open Challenges and Future Work

Table 1 summarizes the main contributions of the literature presented in this document. Mobile M2M communications have the potential to introduce decisive advantages to different fields; however, from literature it is clear they will face several challenges and requirements, many introduced by M2M devices, which are usually constrained in, among others, energy, bandwidth, or processing and memory capabilities.

The support of multitude and diversity of devices, and the traffic volume and traffic pattern generated from them will continue to be important challenges in mobile M2M communications. It will be necessary to support both legacy and new services and devices. Since existing wireless and cellular networks provide higher bandwidth for downlink communications than for uplink, mobile M2M traffic pattern can be a problem for communications if there is not a careful plan of the networks. Traffic volume envisioned for M2M demands for capacity planning. The superposition of Human-based traffic with M2M traffic can expose network limitations in terms of the maximum capacity of network.

In our vision, further research should focus in exploring the mobile M2M gateway concept by seeking ways to improve performance, or reduce the energy and bandwidth consumptions in transmissions, as resource usage efficiency is a common denominator in the literature due to the mass scale envisioned for mobile M2M communications. To the best of our knowledge, techniques to efficiently aggregate and process data at M2M gateways have not been sufficiently studied, and they shall depend on many characteristics of M2M devices, traffic, *etc.* Nevertheless, the concept is being discussed by current standardization efforts, like the aggregating gateway envisioned in MQTT-S or the intermediary node in CoAP, and by literature.

Overheads from handover inefficiency, introduced by mobility, are still a problem in mobile communications and they need to be further mitigated; however, whenever a device can connect to multiple different networks, e.g., WiFi and 3G, the device can exploit the scenario for its advantage, either by using multiple paths and/or the most economic network in terms of resource usage. Future work should also seek to combine techniques, such as transmission scheduling schemes and data aggregation, and explore techniques that effectively reduce the amount of data necessary to be transmitted, such as data compression or data concatenation, to optimize the overall performance.

## 3. Interoperable M2M

Several M2M solutions have been developed to serve a specific business application, which resulted in a dispersion of the technical solutions [40,41]. As a consequence, current M2M markets are highly segmented and often rely on proprietary solutions. But for M2M communications meet the expectations of new business and revenue opportunities while reducing maintenance and resource costs [20], future M2M markets need to be based on industry standards to achieve explosive growth [25]. Additional deployment obstacles include lack of market awareness, technology complexity, initial deployment cost, operator complexity, and operator return on investment concerns [20].

To enable interoperability between M2M services and networks, the European Telecommunications Standards Institute (ETSI) established in 2009 a Technical Committee (TC) focusing on M2M Service level. There are two other reference architectures for M2M: the 3rd Generation Partnership Project (3GPP) - Machine Type Communications (MTC) and IEEE 802.16p M2M, focus in enhancing access and core networks, respectively. These two architectures are complementary to ETSI M2M, and therefore it is possible to combine ETSI M2M architecture with either, resulting in a cellular-centric M2M service architecture [42]. To avoid worldwide market fragmentation and reduce standardization overlap, the oneM2M Partnership Project [43] was created in July 2012 to develop one globally agreed upon M2M specification, initially focusing on consolidating M2M Service Layer standard activities into oneM2M [44]. Most of its current specifications are based on the ETSI M2M Service Layer, therefore we focus on ETSI M2M perspective in the next section.

## 4. ETSI M2M Architecure

The ETSI M2M architecture is currently the reference architecture for global, end-to-end, M2M service level communications, and is being adopted by main European telcos. The system architecture is based on current network and application domain standards, and it is extended with M2M Applications and Service Capabilities layers (SCLs). SCLs are Service Capabilities (SCs) on the Network domain, M2M Device, or M2M Gateway. SCs provide functions to be shared among different M2M applications. The functions of a SCL include, but are not limited to, registration of applications, provision of means for storage, policy-based selection of communication means for information delivery, support for multiple management protocols, or support of remote management of gateways and devices [45].

**Figure 1.**
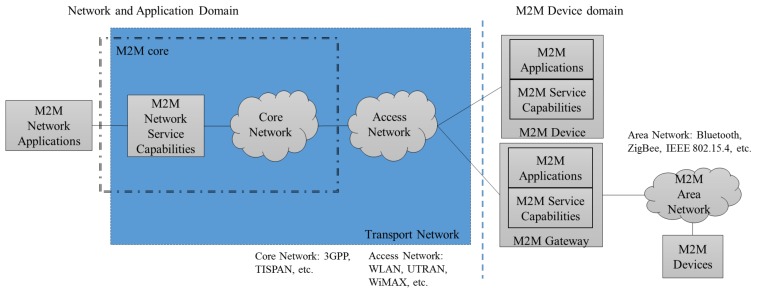
European Telecommunications Standards Institute (ETSI) Machine-to-Machine (M2M) high level system overview.

Figure 1 shows the high level ETSI M2M system architecture as defined in ETSI Technical Standard (TS) 689 [8]. The key entities in M2M are [46]:

M2M Device: a device that runs application(s) using M2M capabilities and network domain functions;
M2M Gateway: guarantees M2M devices interconnection to the network and their inter-operability;
M2M Applications: applications that run the service logic and use Service Capabilities accessible via open interfaces;
M2M Area Network: provides connectivity between M2M devices, compliant and non-compliant with ETSI M2M, and ETSI M2M gateways;
M2M Network and Application Domain: provides connectivity between the M2M gateways and M2M applications;
M2M Network Applications: applications, in the Network and Applications domain, that run the service logic and use Service Capabilities accessible via open interfaces.

The Network and Application Domain is formed by the Access Network, the Transport Network, and the M2M Core. The Access Network provides connectivity between the M2M Device Domain and the Core Network, and the Transport Network provides connectivity within the Network and Application Domain. Satellite, UTRAN, WLAN, UWB, or WiMAX technologies are used in the Access Network. The M2M Core is composed by the Core Network (CN) and M2M Network SCs. The CN provides IP connectivity, interconnections, and roaming capabilities within the M2M Core. Technologies provided by 3GPP or TISPAN can be used in the CN.

The M2M Device domain is formed by M2M devices, M2M Area Networks, and M2M gateways. The M2M devices can connect directly to the Network and Application domain using the Access Network, or they can connect first to an M2M gateway using the M2M Area Network. In the first case, the devices run an M2M application and have an M2M SCL. In the latter, the M2M gateway runs an M2M application and an SCL, and provides access to the Access Network for the M2M Device, acting on its behalf, since the M2M Device has only an M2M Application running, but no SCL, and it is not compliant with ETSI. The Area Network provides connectivity between M2M devices and M2M gateways, and can be built on Bluetooth, UWB, ZigBee, M-BUS, or IEEE 802.15.4 technologies.

The TS also specifies several interfaces not shown in the figure. The interface between an M2M Application in the M2M Device Domain and the M2M SC in the Network and Application Domain is termed mIa. The interface between an M2M Device or M2M Gateway and the M2M SC in the Network and Application Domain is named mId. Finally, the dIa interface is defined between an M2M Application in an M2M Device or M2M Gateway and the M2M SC at the same device.

**Figure 2.**
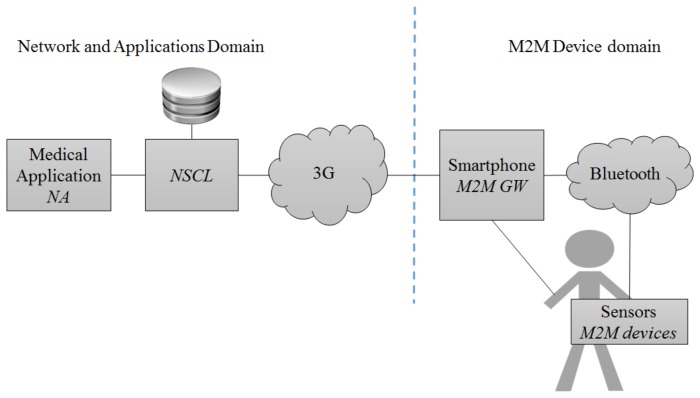
High-level view of the storyboard.

To better illustrate some entities, we describe a storyboard and map it in M2M, similar to [47]. Jonathan is concerned with his well-being. He is an elderly person and needs to control his heart condition. During his daily life he wears a wearable monitoring system that monitors his heart rate. Every morning, he picks up his smartphone, which connects to the wireless sensor to retrieve the measurements. These measurements are stored online, enabling a service that later provides his doctors access to his heart rate history. Figure 2 shows an overview of this storyboard. In an M2M ecosystem, Jonathan, a user, connects his smartphone, acting as an M2M Gateway, to collect information from sensors, M2M devices, over Bluetooth (the M2M Area Network technology) using an M2M Application. The M2M Gateway sends the data using 3G to a Network SCL (NSCL), which main function is to manage the data. In this case, the NSCL stores the data for backup storage purposes and sends the content to a medical Network Application.

The M2M Management functions are all the functions required to manage M2M Applications and M2M SCs in the Network and Applications Domain, and the Network Management functions are all the functions required to manage the access, core, and transport networks [48]. The management functions include performance management, configuration management, fault management and software and firmware upgrading management. M2M Application life cycle management includes installing, removing, and upgrading applications in an M2M Device or Gateway. M2M service management includes configuration management for the M2M SCs in the M2M Device or Gateway. M2M Area Network management includes configuration management for the M2M Area Networks. M2M Device management includes the configuration management of the M2M Device or Gateway.

In ETSI M2M, the M2M service platform is a horizontal middelware, thus applications share common environments, infrastructure, and network elements. To ensure interoperable M2M networks and services, M2M should be agnostic of technologies.

## 5. M2M Communication Models and Paradigms

Before reviewing M2M application protocols used to implement the communication between M2M entities, we discuss here the underlying communication models envisioned for M2M applications and the RESTful and publish-subscribe paradigms. These are the drivers for the protocols that are currently being considered for implementing the communication among M2M entities.

Communication among M2M entities can be categorised in two patterns: event- and polling-based [49]. Polling-based M2M communications follow a request-response communication model. M2M Devices or Applications send requests for specific data, e.g., to actively sample measurements values. Event-based communications are triggered by the occurrence of a particular event, like, for example, the change in value of a variable. M2M Devices or Applications send data to other entities spontaneously, *i.e.*, not in response to a specific request. This pattern is more adequate to M2M application scenarios that require timeliness of reaction upon the occurrence of an event of interest, but can also be used in other scenarios.

Event-based communication causes fewer message transmissions, as there are no explicit requests of information. This message reduction can be of extreme importance when networks and devices are constrained. For example, a service constantly polling a mobile device, like a smartphone and nearby sensors (M2M Devices), about the activity level of an individual can cause unnecessary energy (and bandwidth) consumption for all the unnecessary requests while there is no new value to be reported. A better approach in terms of resource efficiency is to use event-based communications, in which only new activity level values originate message exchange.

ETSI adopts a RESTful architecture style to organise how M2M entities communicate with each other [45]. Representational State Transfer (REST) is a client-server based architectural style created by Roy T. Fielding in 2000 [50].

REST allows contents changing over time. The main concepts in REST are the stateless interactions between clients and servers to manipulate data, and the notion that a distributed application is composed of resources, that each has a particular state, and that each is uniquely addressable. Stateless interactions precludes that every request from a client to a server must contain all of the information necessary for the server to understand the request, and the server cannot use any previously stored context [50]. Stateless communications induce properties of reliability and scalability, since every request can be treated independently. However, REST's statelessness requirement for client-server interactions can be intolerable for constrained wireless devices and networks in mobile M2M communications, either in network bandwidth or energy consumption, due to the required amount of information to be transmitted in every request and response. REST uses CRUD (Create, Read, Update, Delete) operations to manipulate the resources. These operations can manipulate any resource and, therefore, the same architecture can be used by several applications, avoiding the use of dedicated infrastructures. REST foresees the use of intermediaries, or proxies, that perform caching of information to deliver greater scalability.

REST is inherently a request-response based architecture. But, for event-based communications, publish-subscribe is a more reasonable choice. Publish-subscribe is a one-to-many communication paradigm, in which entities, termed subscribers, state their interest in being notified of data/events produced by other entities, termed publishers, at message brokers. This manifestation of interest in events is termed subscription and occurs only once. Publishers transmit to message brokers, and these deliver the message to all the subscribers, whereby both communication entities do not need to be online simultaneously. Subscribers are notified as data/events are produced, reducing the dissemination time and improving the scalability, when compared to request-response. Eliminating the active requests for content, the number of messages, and thus transmissions, is reduced as is the energy consumption of the overall system, which is of extreme importance in scenarios where nodes and networks are resource constrained. This model introduces an additional complexity only in the entity that manages the event notifications, which may be largely compensated by the reduction in the complexity of the nodes.

Time and space decoupling allows greater scalability and flexibility than polling-based communications, and allows a more dynamic topology [51], as publishers and subscribers do not need to be actively participating in the interaction at the same time, and since they do not hold references about each others, each can change their location without being necessary to inform the others.

## 6. M2M Application Protocols

It is impossible to discuss resource efficiency in devices using mobile M2M applications without covering application protocols. M2M application protocols take a fundamental role in communication efficiency: protocol overheads, necessary number of management/control and information messages, reliability, security, *etc.*, all impact the number and size of transmissions and, consequently, the energy and bandwidth consumptions in a mobile device.

The technical plenaries of the oneM2M Partnership Project [52,53] came to an agreement to take into account CoAP, HTTP, and MQTT for communications, strengthening the idea that these protocols are the *de facto* protocols for mobile M2M communications. In the following sub-sections, we describe CoAP [54] and MQTT [55]. We do not review HTTP due to the vast literature already available [56–58]. Although there are other application protocols that can be used for M2M, such as the Advanced Message Queuing Protocol (AMQP) [59], or the Extensible Messaging and Presence Protocol (XMPP) [60], CoAP and MQTT specifically target constrained networks and devices, relying in an effective reduction of protocol overheads.

### 6.1. HTTP and CoAP

CoAP is a lightweight protocol that complies with the REST paradigm [54], and is designed for the use in constrained networks and nodes in M2M applications. In REST architectures, as in HTTP, clients perform operations on resources stored at a server by means of request and responses exchanges. There are four types of requests for the clients:

- GET - gets/retrieves the content of an existing resource;
- POST - creates a new resource;
- PUT - changes/updates the content of an existing resource;
- DELETE - deletes/removes an existing resource.

An Uniform Resource Identifier (URI) is used to identify resources, as in HTTP. CoAP easily translates to HTTP for integration with the Web, while accomplishing specialized requirements such as multicast support, built-in resource discovery, block-wise transfer, observation, and simplicity for constrained environments. CoAP also supports asynchronous transaction support. Like in HTTP, the clients do not need to maintain state, *i.e.*, clients can be stateless [54].

CoAP is conceptually separated into two sub layers: a messaging layer that provides asynchronous messaging services over datagram transport, and a request-response layer that provides handling and tracking of requests and responses exchanged between a client and service side application endpoints. The request-response layer provides direct support for web services. The CoAP request-response semantics are carried in CoAP messages, and a token is used to match responses to requests independently of the underlying messages. Every response message can be returned within an ACK message, that is, piggybacked.

CoAP also supports asynchronous responses, for the cases when the service side knows that it will take long to answer a request. If a client knows at start that an asynchronous response is expected or tolerated, then it includes a Token Option in the message. If the service side knows it might need a longer time to fulfil a request from a client, then it might ask for the client to add a Token option again to that message.

**Figure 3.**
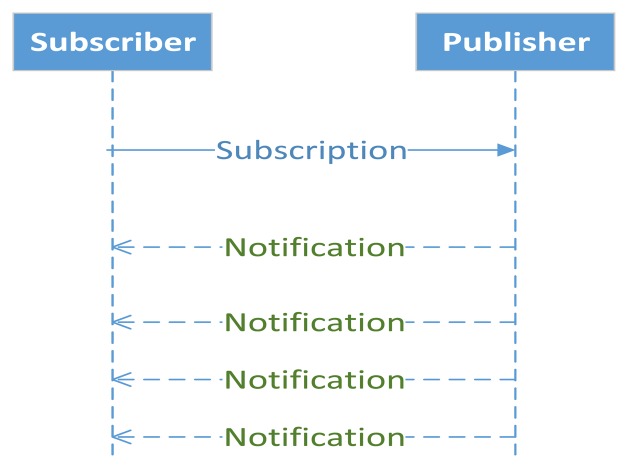
Observer Design Pattern.

The messaging layer implements the publish-subscribe model. This part of the protocol extends the CoAP core protocol with a mechanism for a CoAP client to constantly observe a resource on another CoAP entity, thus termed CoAP observer model [61]. This mode requires that each M2M entity has a client and a server. To use the same terminology as in publish-subscribe, from now on a client is a subscriber and the observation is a subscription. The subscription is made with an extended GET message. With the subscription, each subscriber that has an observation relationship with the event is notified by the publisher where it made the subscription, see Figure 3. In this model, the publisher also acts as a broker. As long as subscribers send acknowledgements of notifications sent in Confirmable CoAP messages by the publisher, the subscriber remains on the list of observers. If the transmission of a notification times out after several attempts or the subscriber rejects a notification using an RST message, then the subscriber is removed from the list of observers. The observer model follows a best-effort approach for sending new representations to subscribers because, if the network is congested or the state changes more frequently than the network can handle, the publisher can skip notifications for any number of intermediate states.

The observer model provides consistency between the actual resource state at the publisher and the state observed by each subscriber, thus keeping the architectural properties of REST. Resource discovery is performed by sending a Confirmable GET request to the resource “well-known/core” at the server.

In order to make CoAP suitable for constrained devices (memory, processing, bandwidth, and energy consumption restrictions), the User Datagram Protocol (UDP) is the preferred transport protocol due to less protocol overheads than the Transmission Control Protocol (TCP), and CoAP's header can be reduced to 4 bytes. CoAP provides two types of application reliability to the delivery of publish messages: a Confirmable (CON) message, where the message is retransmitted if no delivery acknowledgement was received, using a simple stop-and-wait retransmission reliability with exponential back-off for congestion control; and a Non-Confirmable (NON) message, where there is no need to acknowledge the message. There is also a duplicate detection for both Confirmable and Non-Confirmable messages. There are two additional types of messages: Acknowledgement (ACK) or Reset (RST) messages. The ACK message is used to acknowledge CON messages, and the RST message either notifies the other endpoint that a CON message was received but some context is missing, or it is used to cancel subscriptions.

Since UDP is used, the use of multicast IP destination addresses is supported, and can be useful in case of notifications. Security can be implemented by the Internet Protocol Security (IPsec) or Datagram Transport Layer Security (DTLS), although the last option shall be preferred. According to [54], DTLS will introduce at most 13 Bytes overhead per packet, not including initializations. Recent work identified this as a potential problem and there is a proposal for reducing the packet size overheads of DTLS by means of 6LoWPAN header compression saving at least 14% [62].

According to Davis *et al.* [51], CoAP enables high scalability and efficiency through a more complex architecture which supports the use of caches and intermediaries (proxies), similarly to HTTP. The protocol supports the caching of responses in order to efficiently fulfil requests. This caching can be provided by a node in an endpoint or an intermediary. Another important mechanism in the protocol is the proxy functionality. Proxying is useful in constrained networks to improve performance or network traffic limiting, since, for example, proxies are not as limited, in bandwidth or battery, as other nodes. A gateway is considered to be a form of proxy or intermediary.

### 6.2. MQTT

MQTT, developed by researchers at IBM, is a lightweight broker-based publish-subscribe messaging protocol designed to be open, simple, lightweight, and easy to implement [55]. The authors claim that MQTT's characteristics make it ideal for use in constrained environments, where, for example, the network is expensive, has low bandwidth or is unreliable, or when it runs on embedded devices with limited processing or memory capacities. MQTT does not comply with REST.

MQTT is an asynchronous protocol. Some MQTT messages contain a variable header, present after the fixed header and before the payload, that contains, for instance, the protocol name, the protocol version, and flags. There are 14 different message types defined in MQTT, including CONNECT, PUBLISH, SUBSCRIBE, UNSUBSCRIBE, DISCONNECT, and PINGREQ and PINGRESP. Several of these messages have dedicated acknowledgement messages. For example, if an MQTT subscriber wants to subscribe to a topic, the subscriber sends a SUBSCRIBE message, and waits for the correspondent SUBACK response from the broker (server).

Even though the use of MQTT implies the use of a reliable connection oriented transport protocol, like TCP, MQTT supports three types of application reliability to the delivery of publish messages:

- Level 2 - deliver message exactly once, which guarantees that messages arrive exactly once;
- Level 1 - deliver message at least once, which guarantees that messages arrive, but duplicates may occur;
- Level 0 - deliver message at most once, where messages arrive according to the best efforts of the underlying TCP/IP network, which means that message loss or duplication, introduced by software or other causes, can occur.

These options are selected in the QoS flag present in the header of each MQTT message.

The protocol has a small header whose fixed-length is just 2 bytes, and protocol exchanges are minimized to reduce network traffic. The standard does not specify security mechanisms, therefore IPSEC or TLS can be used.

MQTT for Sensor Networks (MQTT-S) [63] is an extension of MQTT. MQTT-S is optimized for the implementation on low-cost, battery-operated devices with even more limited processing and storage resources, such as wireless sensor devices. While MQTT is based on the TCP/IP stack, MQTT-S operates on any network technology that provides a datagram service, like UDP. MQTT-S is aimed at minimizing network bandwidth and device resource requirements while targeting reliability. Additionally, an MQTT-S gateway can be integrated in the broker to translate between MQTT-S and MQTT.

**Figure 4.**
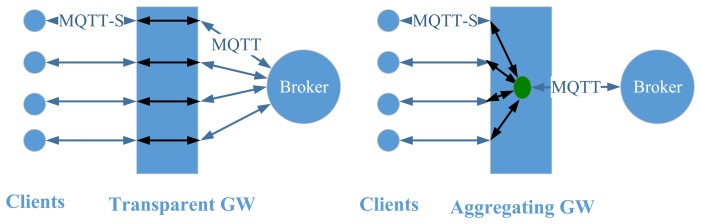
Transparent and Aggregating Gateways.

Figure 4 illustrates the concepts of transparent and aggregating gateway. A transparent gateway sets up and maintains an MQTT connection to the MQTT broker for each connected MQTT-S client. The gateway only performs translation between the two protocols. The implementation of this type of gateways is simpler than the implementation of an aggregating gateway. The use of an aggregating gateway requires only one MQTT connection to the broker. While connections at the transparent gateway are end-to-end (client to broker), connections with an aggregating gateway end at the gateway (client to gateway) that then decides which information will be given further to the broker [63]. Therefore, there is a trade-off between complexity and scalability of the gateways' implementation.

### 6.3. Evaluation and Comparison

The functionality of CoAP has been experimentally validated. The main functions of CoAP, working over UDP, observe and discovery, and its interworking with HTTP have been verified by Bormann *et al.* [64], and the feasibility of an ETSI-compliant complete end-to-end system using CoAP is demonstrated in [48]. The transport of CoAP over Short Message Service (SMS) [65] has also been already implemented and evaluated in [66]. A prototype web platform, which integrates a CoAP Wireless Sensor Network (WSN) with an HTTP web application and allows a user to visualize WSN measurements in the web browser is described in [67], demonstrating transparent cross-protocol resource access by means of an HTTP-CoAP proxy. There is also an open source implementation of CoAP written in C often used in the literature [68,69], Libcoap [70].

There are two open source MQTT implementations commonly used in experimental validations, Mosquitto [71] and Paho [72]. The original developers of MQTT-S implemented an MQTT-S client and gateway and validated them experimentally, having several devices forward packets received from a wireless network to a gateway [73]. Apart from small limitations referred in the paper, the gateway was considered to be fully functional. However, further testing with larger number of devices is necessary to evaluate the protocol performance.

Table 2 provides a comparison of the main features of CoAP and MQTT. Although CoAP's header is twice as large as MQTT's header, both can be considered very small when compared to application protocols not specific to constrained devices, such as HTTP. The transport layer protocol is a decisive feature in the performance of each protocol. An experimental comparison between CoAP running over UDP and MQTT running over TCP, using libcoap and mosquitto implementations, respectively, is provided in [68]. As expected, due to inherent transport layer overheads, they concluded that MQTT consumes higher bandwidth than CoAP for transferring the same payload under same network conditions. From these results, we expect MQTT-S to provide a similar performance to CoAP since it also uses UDP, but this comparison has not been performed so far.

CoAP over UDP has also been compared with HTTP over TCP and UDP. The results showed that UDP based protocols perform better for constrained networks (both CoAP and HTTP) than TCP based protocols, due to using lower number of messages when retrieving resources [69]. However, it is preferred to use CoAP over UDP rather than HTTP over UDP, since the first provides reliability mechanisms. For a fair comparison of application protocols, they should use the same transport protocol, same communication model (in this case, publish-subscribe), and similar parameter values whenever possible. Such performance evaluations have not been made available so far in M2M contexts.

A further difference between CoAP and MQTT is their application reliability: CoAP provides two levels of application reliability, correspondent to Level 0 and Level 1 in MQTT/MQTT-S, which have yet another one, Level 2. The reliability mechanisms of both protocols employ a fixed retransmission time-out. This parameter has a direct impact on protocol performance, namely packet delivery ratio and duplicated publications when using Level 0 or Level 1 of both protocols. The increase of retransmission time-out leads to higher packet delivery ratio, and the effect is more visible as the number of publisher nodes increase. Still, overall and for similar configurations, CoAP achieved better packet delivery ratio than MQTT-S in Omnet++ simulations, mainly due to differences in the publication discipline [51]. CoAP's non-persistent publication discipline gives priority to sending new publications while MQTT-S attempts to retransmit old ones. CoAP abd MQTT allow messages to be sent and received asynchronously, but only CoAP supports synchronous messaging. Finally, only CoAP provides a request-response protocol compliant with REST concepts.

Both CoAP and MQTT-S support the use of UDP, intermediary nodes, gateways, to perform requests and responses/relay messages on behalf of other nodes, do caching, aggregation, *etc.* So, further studies, deployments and field trials need to be conducted to assess their performance, especially in constrained devices and networks, and when a large number of devices is present.

MQTT does not provide service discovery. The performance of CoAP service discovery is discussed in [74]. In summary, CoAP discovery protocols show better performance in terms of overhead than DNS-based discovery protocols, since it was designed aiming at resource efficiency for constrained devices and networks. Furthermore, based on measurements and functionalities, the authors claim that CoAP's resource discovery allows a more efficient and richer set of mechanisms to perform lookups than DNS-based protocols.

## 7. Smartphones as Mobile M2M Gateways

A potential use case for mobile M2M communications is its application in healthcare for remote monitoring of patient vital signs, e.g., activity level, blood pressure, heart rate, temperature, either for ambulatory monitoring of chronic conditions, or for prophylactic reasons, in order to minimize the number of visits to the doctor [11,26]. In the latter scenario, there are wearable sensors that continuously collect physiologic information and send it to a remote service to be processed and acted upon.

Table 3 shows some characteristics of representative traffic originated in current healthcare sensors for remote vital sign monitoring, as presented in [75]. Different physiologic variables have different sampling rates and number of channels. The number of channels reflects the number of different locations of the body from where we are sampling the variables.

Although it is not expected that an individual carries an Electroencephalogram (EEG) sensing device the entire day, we can envision the application of such devices in a medical facility, where it might be necessary/useful for continuous remote monitoring. As shown in the table, the EEG is expected to produce a higher data rate than, for example, the body temperature variable.

Sensors often forward the collected data to a nearby mobile gateway using short-range wireless technologies, like Bluetooth, for energy saving. This mobile gateway can collect data from only one or several sensors, and forward it to destination service. Instead of simply forwarding information as it arrives, the mobile gateway can collect, aggregate, and eventually process information to reduce the number of transmissions and eventually the amount of data, and energy consumed in undesired network and protocol overheads. Transmitting the highest possible amount of information in the least number of packets improves resource utilization efficiency. However, different sensor data has different granularity, timeliness requirements, or tolerance to packet loss. For example, EEG data is more important than a room temperature, and, therefore, it will have stricter delivery requirements.

Just a single blood pressure sensor with a sampling frequency of 120 Hz, needing 16 bits to send the data, results in 1920 bit per second (Table 3), 6.9 Mbit per hour, and 165 Mbit per day, excluding any communication protocol overheads. The more sensors the mobile gateway has to support, the more data needs to be collected and forwarded. This situation introduces a considerable challenge to the mobile gateways in terms of energy consumption, since they have limited battery life, and can have different time intervals between battery replacement/recharge.

Smartphones are an ideal choice to play the role of a mobile M2M gateway in healthcare applications, as they are largely available to the population, are less resource constrained than specialized sensors, and have various connectivity alternatives. Although M2M communications have been thoroughly addressed for healthcare applications [13,15,26,38,39], most of the literature for healthcare still focus on BANs of sensors, and do not explore scenarios potentiated by the use of smartphones.

The use of smartphones for transmission of sensing and monitoring data can lead to undesirable battery depletions, since users use their smartphones mainly for other purposes, mostly personal communication and browsing. For the user, it might be desirable to recharge the smartphone only during the night when the smartphone is no longer needed, which means that the collection, processing and forwarding of data should not exceed a certain amount of energy consumption during the day, in order for the battery to last at least 12 h, e.g., from 08:00 to 20:00. Several studies have been conducted on recharge patterns [76–78]. Battery recharges are triggered either by the current battery level, or by the context, which includes location and time of day. Users that recharge battery based on battery levels, notice differences in the recharging cycle of the phones, and tend to be irritated by the increase of energy consumption [78]. Results from [76] show two major charging schedules: one between 18:00 and 20:00, and another charging schedule between 01:00 and 02:00, and the majority of the charging durations are 2 h or less or 14 h or more. From interviews in [77], persons usually recharge once their mobile equipment at the office, at home, or at night. Since user behaviour in terms of battery recharge may vary a lot [76], we consider that, for user convenience, phones are recharged only once per day, at night, and they need to operate from 08:00 to 20:00.

**Figure 5.**
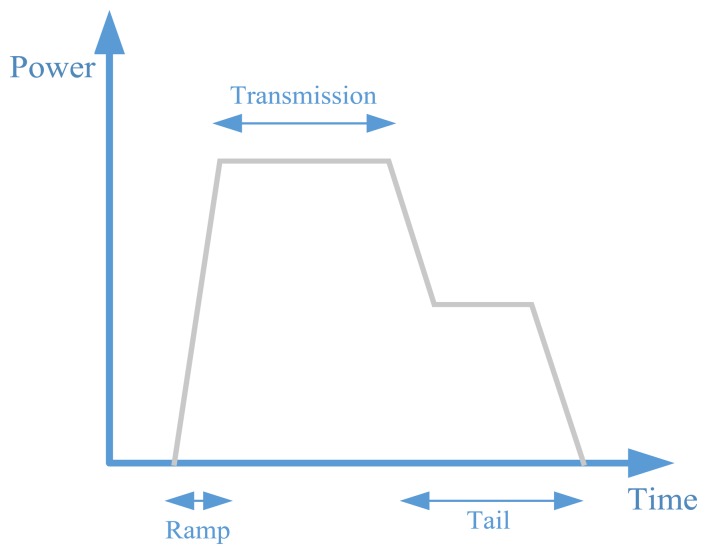
Common three stages of a 3G transmission: the ramp and the tail are transitions to and from high-power states where the actual transmission takes place. Based on [79].

We seek to understand the feasibility of using a smartphone as an M2M gateway in terms of the energy consumption for forwarding data using a cellular network. The ubiquity and high data rates make cellular networks the most common choice for mobile M2M applications. As sensor data can be obtained using different short-range low-powered technologies, for the sake of simplicity, we exclude the data collection from the analysis. Nevertheless, we stress that obtaining the sensing data in an optimal way in terms of energy consumption is of extreme importance. For this study, we use the models for the average energy consumed for uploading data from a smartphone using a 3G network, obtained from [79]. Transmissions in 3G can be defined in 3 periods: the ramp, the transmission, and the tail, as can be seen in Figure 5. The ramp and the tail are transitions to and from high-power states where the actual transmission takes place, respectively. The time duration of the ramp is relatively small when compared to the actual transmission and the tail. However, the tail can last up to 12.5 s [79]. Still, if the data to be transmitted is large, or if the interval between successive transfers is small, the tail and the ramp do not impact the energy consumption significantly [79] when compared to the actual transmission.

The energy consumption model takes as inputs: the necessary energy to transfer *x* KB, *R*(*x*) = 0.025 × + *x* 3.5 J, which already includes the energy consumed in scanning and association with the network; *M* = 0.02 J/Sec as a maintenance energy value to keep using the interface; and the energy consumed during the tail time, *E* = 0.62 J/s, where the maximum time in this period is 12.5 s. Therefore, for example, transmissions of 100 KB with intervals of 50 s between consecutive transmissions (the device becomes idle after the tail) will consume 0.025 × 100 + 3.5+ 0.62 × 12.5 +0.02 × 50 = 12.25 J.

We assume that regular user activity depletes the battery in 100 mAh, an intermediate value between the 10 and 250 mAh presented in [80]. The average battery for a middle range priced smartphone, like the Samsung Galaxy Nexus [81], has a capacity of 1750 mAh at a voltage of 3.6 V. Therefore, assuming the battery voltage remains constant during normal operation, the smartphone should last at least 17 h when not additionally functioning as a mobile M2M gateway.

**Figure 6.**
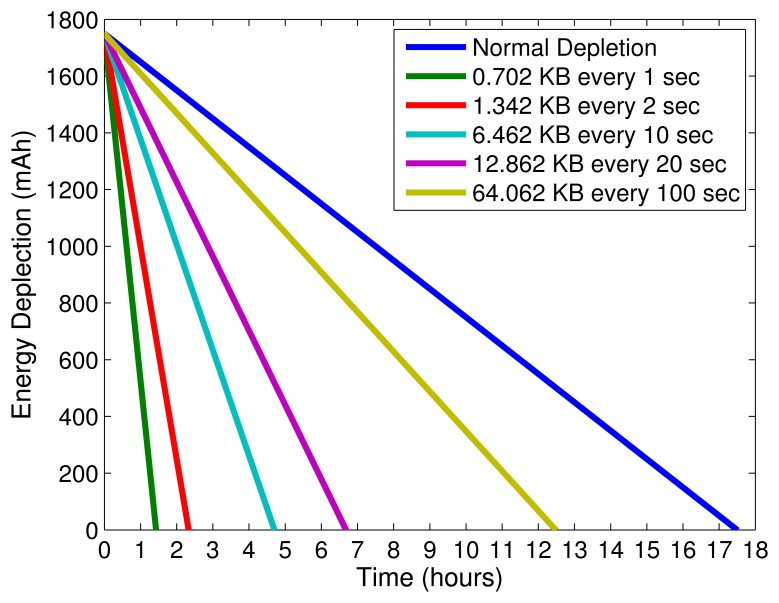
Battery consumption for different transmission schemes using 3G.

Please now consider the scenario where a smartphone collects data from several sensors to be forwarded, as depicted in our storyboard and in Figure 2. In this case, we assume that there is only one sensor collecting the heart rate from an individual and forwarding the data to a smartphone. From Table 3, we can conclude that, in each second, the sensor generates approximately 0.64 KB of data. The smartphone has to transmit this data through 3G and needs to append protocol headers and likely execute security mechanisms. We assume the use of CoAP application protocol, no header compression, and that each transmission includes a fixed overhead of 62 B to the useful data: 10 bytes for a common CoAP confirmable request [54], 13 bytes for DTLS per packet [54], 8 bytes for the UDP header, 20 bytes for the IP header, 2 bytes for the PDCP header, 5 bytes for the RLC and MAC Headers, and 4 bytes for CRC [82]. The data can be forwarded as 0.702 KB every second, 1.342 KB every two seconds, 6.462 KB every 10 s, and so forth, depending on the aggregation chosen. Figure 6 depicts the battery depletion of the smartphone for different transmission rates using 3G. Due to the scale in hours of the *x* axis, it is not possible to visualize the changes in slope when transmissions occur. The results show that only for larger aggregations of transmitted data, 64 KB every 100 s, we can achieve a full day, e.g., 08:00 – 20:00, of battery operation. We conclude that frequent transmissions of small size data will have an undesired effect in the expected depletion time of a smartphone's battery in mobile M2M communications scenarios. In order to use smartphones as M2M gateways, it is a good advice to maximize the collection of data necessary to be forwarded from nearby sensors, and maximize the intervals between transmissions. More research is required to devise energy efficient transmission methods that enable the use of smartphones as mobile gateways. Techniques such as transmission scheduling, header compression, data aggregation, or leverage heterogeneous networks can enhance smartphones' capabilities acting as gateways while easing their energy consumption problems.

## 8. Conclusions

Mobile M2M communications are currently receiving attention from the academia due to its potentiality in ubiquitous applications, like mobile healthcare, telemetry, or in intelligent transport systems, and also due to the emergence of IoT paradigm. In this paper, we reviewed the standardization efforts and application protocols, and draw considerations on the impact of their use in constrained devices.

M2M communications in cellular and wireless networks will face several challenges, and we reviewed literature that focus on performance evaluation and improvement, either in terms of delay or resource usage efficiency. Future work should focus in exploring the M2M gateway and mechanisms to efficiently collect and aggregate data while attaining the time requirements of data, to reduce energy and bandwidth consumptions, as resource usage efficiency is a common denominator in the literature due to mass scale envisioned for mobile M2M communications. Techniques such as data aggregation, concatenation, or compression mechanisms might be useful to reduce both non-useful and useful data. Overall, resource usage efficiency in mobile M2M communications is still an open research area, and further studies on the impact of multitude and diversity of devices and traffic in the performance of communications are necessary.

Finally, we performed a preliminary study on the feasibility of using smartphones as M2M gateways that collect and aggregate information from sensors. We concluded that, in order for their use to the feasible in terms of a normal depletion time of a smartphone's battery, it is a good advice to maximize the collection of data necessary to be forwarded from nearby sensors, and maximize the intervals between transmissions.

## Figures and Tables

**Table 1.** Summary of survey of literature and challenges presented in the document.

**Contributions**		**Reference**
Human-based *vs.* M2M Communications		Lien *et al.* [21], Laya *et al.* [17]
Technical Challenges		Wu *et al.* [25], Zhang *et al.* [12], Chen [15], Lien *et al.* [21]
Requirements		Lu *et al.* [22], Zhang *et al.* [12], Lien *et al.* [21]
Applications	Healthcare	Chen [15], Dawson-Haggerty [38], Marwat *et al.* [26], Fan *et al.* [13], Jung *et al.* [39]
	Vehicles	Booysen *et al.* [37]
	Airlines	Plass *et al.* [34]
Mobility		Booysen *et al.* [37], Lee *et al.* [35], Kellokoski *et al.* [36]
Performance Evaluation	QoS provision	Marwat *et al.* [26]
	Throughput	Marwat *et al.* [26]
	Interference	Costantino *et al.* [27]
	Access Delay	Lien *et al.* [30], Gallego *et al.* [23]
Channel Access	Energy Efficiency	Gallego *et al.* [23]
	Latency	Zhou *et al.* [29]
	QoS provision	Zhang *et al.* [12]
Transmission Scheduling Schemes	Delay	Yunoki *et al.* [32]
	Power Consumption	Paulset *et al.* [33]
Data Aggregation	Delay	Lo *et al.* [31]
	Packet Collisions	Matamoros *et al.* [24]
	Throughput	Lo *et al.* [31]
Mobile M2M Gateway		Wu *et al.* [25], Zhang *et al.*[12]

**Table 2.** Comparison between main features of Constrained Application Protocol (CoAP) and Message Queuing Telemetry Transport (MQTT).

	**CoAP**	**MQTT**
**Communications Model**	Request-Response, or Pub-Sub	Pub-Sub
**RESTful**	Yes	No
**Transport Layer Protocol**	Preferably UDP; TCP can be used	Preferably TCP; UDP can be used (MQTT-S)
**Header**	4 Bytes	2 Bytes
**Number of message types**	4	16
**Messaging**	Asynchronous and Synchronous	Asynchronous
**Application Reliability**	2 Levels	3 Levels
**Security**	IPSEC or DTLS	Not defined in standard
**Intermediaries**	Yes	Yes (MQTT-S)

**Table 3.** Characteristics of representative traffic originated in current healthcare sensors.

**Parameter**	**Data Rate (in bps)**	**Sampling Frequency (in Hz)**	**Bits Per Sample Number of Channels**
Body Temperature	2.4	0.2	12 1
Blood Pressure	1920	120	16 1
Cardiac Output	640	40	16 1
EEG	98,304	256	16 24
